# Glass Transition Temperatures and Thermal Conductivities of Polybutadiene Crosslinked with Randomly Distributed Sulfur Chains Using Molecular Dynamic Simulation

**DOI:** 10.3390/polym16030384

**Published:** 2024-01-30

**Authors:** Tannaz Alamfard, Tommy Lorenz, Cornelia Breitkopf

**Affiliations:** Chair of Thermodynamics, Institute of Power Engineering, Faculty of Mechanical Engineering, Technical University Dresden, 01069 Dresden, Germany

**Keywords:** degree of crosslinking, equilibrium molecular dynamic simulation (EMD), Green–Kubo method, thermal conductivity, glass transition temperature, autocorrelation function, polybutadiene

## Abstract

The thermal conductivities and glass transition temperatures of polybutadiene crosslinked with randomly distributed sulfur chains having different lengths from mono-sulfur (S1) to octa-sulfur (S8) were investigated. The thermal conductivities of the related models as a function of the heat flux autocorrelation function, applying an equilibrium molecular dynamic (EMD) simulation and the Green–Kubo method, were studied for a wide range of temperatures. The influence of the length of sulfur chains, degree of crosslinking, and molar mass of the crosslinker on the glass transition temperature and final values of thermal conductivities were studied. First, the degree of crosslinking is considered constant for the eight simulation models, from mono-sulfur (S1) to octa-sulfur (S8), while the molar mass of the sulfur is increases. The results show that the thermal conductivities of the crosslinked structure decrease with increasing temperature for each model. Moreover, by increasing the lengths of the sulfur chains and the molar weight of the crosslinker, thermal conductivity increases at a constant temperature. The MD simulation demonstrates that the glass transition temperature and density of the crosslinked structure enhance as the length of the sulfur chains and molar mass of the sulfur increase. Second, the molar weight of sulfur is considered constant in these eight models; therefore, the degree of crosslinking decreases with the increase in the lengths of the sulfur chains. The results show that the thermal conductivities of the crosslinked structure decrease with the increase in the temperature for each model. Moreover, by increasing the lengths of sulfur chains and thus decreasing the degree of crosslinking, the trend in changes in thermal conductivities are almost the same for all of these models, so thermal conductivity is constant for a specific temperature. In addition, the glass transition temperature and density of the crosslinked structure decrease.

## 1. Introduction

The advancement in computer technology has made molecular dynamic (MD) simulation an essential component of scientific investigations, coordinating theoretical and experimental studies [1,2,3]. MD simulation can display both the internal mechanism of macroscopic experiments and provide the microscopic evolution process at the atomic scale [4,5]. It has been employed extensively in the disciplines of composite materials [6], the petrochemical industry, and biomedicine [7,8], specifically in the polymer field. There are many unpredictable elements [9], and therefore it is essential to apply suitable polymer models and simulation methods in these fields [10].

MD simulations need both a molecular structure model and an appropriate force field. The development of computational models for crosslinked model structures has been investigated by several authors. The early efforts dating back to the 1980s and 1990s were primarily centered on the reaction kinetics of crosslinking procedures applying lattice [11] along with nonlattice [12] Monte Carlo models with regards to the polymerization degree, molecular weight dispersion, polydispersity, etc. The topological details of the system were not taken into consideration, and the majority of these investigations were essentially mathematical [13]. Consequently, significant information regarding mechanical and thermodynamic aspects was not addressed in an appropriate way. Following this, utilizing molecular dynamics simulations, several researchers examined the development of crosslinked structures containing topological details by applying several kinds of crosslinking methodologies. In particular, Doherty and colleagues developed PMA systems applying lattice-based simulations combining the polymerization molecular dynamics technique [14].

It should be mentioned that in recent years, uncrosslinked polybutadiene (PB) has been the subject of many simulations. Theodorou and colleagues have performed atomistic simulations for the polybutadiene isomers cis-1,4-PB and 1,2-PB, applying the end-bridging Monte Carlo technique and the molecular dynamics approach [15,16]. Narros et al. used completely atomistic MD and neutron diffraction to study the short-range order in 1,4-PB [17]. Maurel et al. have created a mesoscale model for investigating the dynamic and static characteristics of PB melt in a few articles [18,19]. Additionally, these researchers studied how cis-1,4-PB chains interact with a silica surface [20]. To determine the glass transition temperature of uncrosslinked cis- and trans-1,4-PB, atomic MD simulation has been used [21]. The main subject of all of these investigations is linear macromolecules. Accordingly, the outcomes of the current research can be regarded as an expansion of these simulations to crosslinked PB structures.

In order to create crosslinked components with thermal, chemical, and mechanical characteristics that are appropriate for a wide range of applications, the most popular method used in the rubber industry is to vulcanize polydiene elastomers by heating them with sulfur, accelerators, and other additives [22,23,24,25], and the most well-known application of this method is the manufacturing of pneumatic tires. In the process of vulcanization, sulfur interacts with the chains of elastomers to create linkages of various lengths, including pendant chains, mono- and disulfidic bonds, and cyclic sulfides. Long polysulfidic linkages are often created early in the vulcanization process; afterwards, desulfuration and breakdown by heat may take place, producing shorter sulfidic connections, altering the degree of crosslinking, and changing the main chains. Accelerators and activators are used to speed up the vulcanization procedure, make rubber processing simpler, and enhance the qualities of the final vulcanized material. Accelerators, specifically, have the ability to break down sulfur chains, which results in less demand for sulfur during the crosslinking process. Moreover, the formation of shorter sulfur fragments results in smaller sulfide linkages in rubber [26,27].

In general, by raising the accelerator/sulfur (A/S) ratio, more sulfur linkages with shorter lengths are formed, and the vulcanization reaction’s effectiveness is enhanced [22,25,26,28,29]. Temperature is another key factor that influences the crosslinking procedure through vulcanization. Specifically, it has been discovered that raising the temperature reduces the crosslink density in natural rubber (NR) during accelerated sulfur vulcanization, reducing the polysulfidic bridges and distributing the di- and monosulfidic bridges [30,31]. The crosslinking procedure increases the polymer’s mechanical characteristics, thermal conductivity, wear resistance, and creep resistance [32,33].

Both amorphous polymers and the amorphous component of crystalline polymers reflect the glass transition behavior when these polymers move from a glassy state to a rubbery phase. According to theory, when polymers go through the glass transition, their specific heat capacity, thermal expansivity, movements of molecular chains, and other characteristics suddenly rise [34,35,36]. Over the past few years, numerous researchers have used MD modeling tools to conduct investigations on polymer systems. Nevertheless, the majority of these investigations used coarse-grained simulations, which often at least largely ignored the chemistry and interactions of the actual system [19,37,38].

The use of entirely atomistic models in molecular simulations of crosslinked polymers is still uncommon. To the best of our knowledge, the majority of studies did not focus on the impact of crosslinking densities on the structure and glass transition temperature (*T*_g_) of these complex structures, instead concentrating on small model systems or entirely crosslinked epoxy structures [39,40]. Additionally, a variety of techniques have been employed in simulations using molecular dynamics in an effort to determine the glass transition temperature [41,42]. One of the most crucial characteristics of polymers is the glass transition temperature, which is still a subject of contention in general. The glass transition indicates the range for both application and temperature for manufacturing [43].

Therefore, it would appear to be very important from both a theoretical and practical standpoint to look at how chemical crosslink density affects the structure and characteristics of crosslinked polymers. In our previous studies, the influence of both uniform and random distributions of di-sulfur crosslinked with PB on the thermal conductivity was investigated by polymeric model structures with similar and variable degrees of crosslinking [44]. However, through the application of completely atomistic computer simulations, the current work seeks to discover how the molar weight of the crosslinker, the degree of chemical crosslinking, and the length of crosslinker chains from mono-sulfur to octa-sulfur affect the glass transition temperature and thermal conductivity of chemically crosslinked PB. It allows us to comprehend the structure–property correlations of these intricate structures on an atomic level. The glass transition temperature was then investigated using constant number of particles, pressure, and temperature (*NPT*) MD simulations on chemically crosslinked PB at various temperatures.

## 2. Methods and Theoretical Formulations

### 2.1. Set-Up of Simulation

#### 2.1.1. Mechanism of Chemical Bond Formation and Crosslinking

This method is used to form crosslinking bonds between pairs of atoms of crosslinker material and matrix during the crosslinking process while a simulation is conducted under specific conditions. This approach can be used to simulate the creation of a percolation network and the crosslinking of polymers, etc. Once the bond is established, the connection will remain permanently in place during the next steps of the simulation. In addition, the formation of a bond can also produce new angles, dihedrals, and improper force fields for the new configuration that the bond is a component of.

The possibility of a bond forming between two atoms, designated as *i* and *j*, is indicated if they are within a particular distance (*R*_min_), there is not already a bond between them, and both atoms *i* and *j* have not reached their respective maxbond requirements (explained below). Throughout the simulation, a check is carried out periodically at specific intervals to determine if a new bond may form. These intervals, known as specific time steps, need to be defined in the simulation settings.

**Figure 1:**
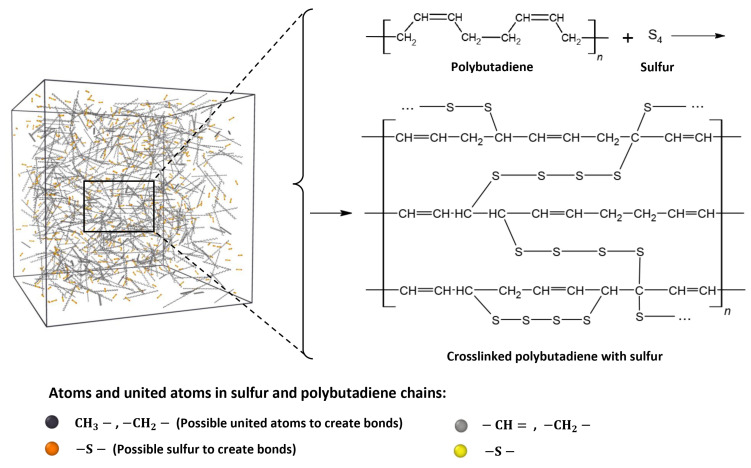
Mechanism of chemical bond formation and crosslinking of PB with tetra-sulfur (S4).

An atom may have numerous potential bond partners if other atoms are nearby. Each atom examines its list of potential bond partners and designates the one that is nearest to it as its “sole” bond partner. After that, the bond between atoms *i* and *j* is “eligible” to form if atom *i* has atom *j* as its sole partner and atom *j* has atom *i* as its sole partner. It should be mentioned that according to these principles, an atom may only be a component of one newly formed bond at a certain time step. It additionally indicates that if atom *i* selects atom *j* as its sole partner, but atom *j* selects atom *k* as its sole partner (since *R*_jk_ < *R*_ij_), then atom *i* cannot establish a bond at this time step, despite the fact that it has other possible bond partners [45]. As has been illustrated in Figure 1, for instance, CH_2_ can be considered as a united atom *i*, and S (Sulfur atoms at the head and tail of tetra-sulfur (S4) chain) can be considered as atom *j* in this method.

#### 2.1.2. Simulation and Creation of Model Systems

**Figure 2:**
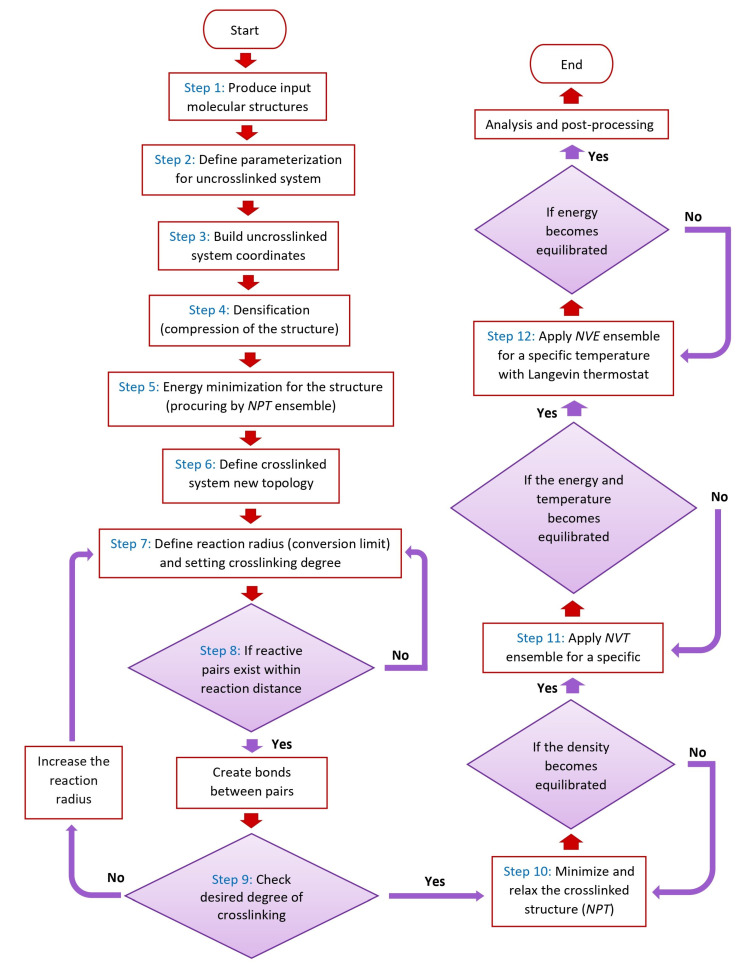
Flowchart that displays crosslinking simulation process and application of ensembles.

As indicated in step 1 of Figure 2, the initial crosslinked model system comprises two materials, matrix and crosslinker, with each material being created separately. Since the essence of the crosslinking reaction is the formation of new chemical bonds, probable atoms (or united atoms), which are capable of creating bonds in both matrix and crosslinker chains, are labeled using particular new names in this step.

Then, the process of parameterization for defining specific topologies, including masses, charges, and force fields, is performed in step 2 of Figure 2 for the uncrosslinked framework. The coordination of the structure, which is made up of a predetermined number of matrix chains and crosslinker chains, is created by reproducing and distributing chains within a cubic cell in step 3. Following that, densification and procure equilibration are conducted in step 4 to achieve a more realistic or desired density for the simulated polymeric material.

After densification, the system might be in a high-energy state due to the arrangement of atoms and molecules; thereby, in step 5, energy minimization with the *NPT* ensemble provides a context to optimize the molecular framework by adjusting bond lengths, bond angles, and dihedral angles to minimize potential energy and ensure a chemically feasible configuration.

After the crosslinking process, new bonds will be formed, and the configuration of the crosslinked polymeric framework will change. Subsequently, the potential interactions of the new polymeric configuration also change. Therefore, new force field parameters need to be adopted, which comprise new bonds and masses, for the newly formed framework in step 6 before the crosslinking process. Furthermore, the specific reaction radius (*R*_min_) and conversion limit, as illustrated in Figure 1, along with setting the condition for the degree of crosslinking, are accomplished in step 7. Then, after running the simulation in step 8, to create bonds, if reactive pairs exist within the reaction distance (*R*_min_), bonds will be created between the polymer and crosslinker; consequently, the degree of crosslinking must be checked in step 9.

The process of forming bonds in a crosslinked structure will cause abrupt changes in the configuration and thus potential energy of the system. The equilibration enables the system to stabilize and ensures a starting configuration that is more realistic. In order to reach the necessary density, the final crosslinked structure is equilibrated with an *NPT* ensemble in step 10 at a certain temperature and pressure if the desired level of crosslinking was accomplished in step 9 of the process.

If the density is equilibrated at the specific temperature, the *NVT* ensemble will be applied to the structure at the related temperature to stabilize the temperature in step 11. If the temperature becomes equilibrated, in order to obtain the thermal behavior of the framework, the *NVE* ensemble with the Langevin thermostat at the associated temperature would be applied to the framework in step 12.

### 2.2. Green–Kubo Method and Heat Flux Formulation

In this paper, the equilibrium molecular dynamic (EMD) approach has been used to examine the heat conductivity of PB crosslinked with sulfur. The Green–Kubo method is one way that is employed here to extract the thermal conductivity; it is based on the fluctuation–dissipation principle and involves correlating the heat flux in each direction with the thermal conductivity (*K*) [46,47]. The following are the equations for thermal conductivity in the *x* direction:

(1) $$K=\frac{V}{k_{B}T^{2}}\int_{0}^{\infty }\left\langle J_{x}\left(t\right)J_{x}\left(0\right)\right\rangle dt\;,\;$$

so that $J_{x}$, $\left\langle J_{x}\left(t\right)J_{x}\left(0\right)\right\rangle$, *V*, *T* and $k_{B}$ are, respectively, the heat flux along the *x* direction, the heat flux autocorrelation function (HFACF), the volume of the framework, the temperature of the system, and the Boltzmann constant. The average of the thermal conductivities along three directions (*x*, *y*, and *z*) must typically be calculated in order to determine the thermal conductivity of an isotropic system. The average thermal conductivities along the *x*, *y*, and *z* directions are thus obtained in the present research through the addition of a factor of three to the denominator of the Green–Kubo equation.

(2) $$K=\frac{V}{{3k}_{B}T^{2}}\int_{0}^{t_{c}}{\left\langle J\left(t\right)\cdot J\left(0\right)\right\rangle }_{t_{s}}dt\;,$$

In the aforementioned formula, $t_{s}$ is the average time frame in which the ensemble for obtaining the HFACF is built up. ***J*** indicates the heat flux vector, and $t_{c}$ is the finite correlation time in which the integration is carried out. There are two methods that can be applied for calculating the heat flux vector [48]. The most typical formula for obtaining the heat flux vector is as follows:

(3) $$J=\frac{1}{V}\frac{d}{dt}\sum_{i=1}^{N}r^{i}e^{i}\;,$$

The terms $r^{i}$, $e^{i}$, and *N* in the expression above, respectively, indicate the position vector of atom *i*, the total energy of atom *i*, and the total number of atoms in the system. The amount of kinetic energy and potential energy of the associated atom can be added to determine the energy of each atom.

(4) $$e^{i}=\frac{1}{2}m^{i}{\left|\upsilon^{i}\right|}^{2}+U^{i},$$

In which $e^{i}$, $m^{i}$, $\upsilon^{i}$ and $U^{i}$ indicate total energy, mass, velocity, and potential energy related to atom *i*.

In the simulations, the interaction potential energy will be employed entirely to define the total potential energy of atom *i* $\left(U^{i}\right)$. Both bonded and non-bonded interactions make up these interaction potential energies. By combining all of the following potential interactions, the overall potential energy is determined: pair interactions $\left(U_{Pair}\right)$ associated with van-der-Waals potential energy, coulomb interactions $\left(U_{Coulomb}\right)$, bond interactions $\left(U_{Bond}\right)$ connected with covalent bonds, angle interactions $\left(U_{Angle}\right)$, and dihedral interaction $\left(U_{Dihedral}\right)$ along with improper interactions $\left(U_{Improper}\right)$, as can be observed in the subsequent equation,

(5) $$U=U_{Pair}+U_{Coulomb}+U_{Bond}+U_{Angle}+U_{Dihedral}+U_{Improper}\;,$$

A certain number of atoms participate in each interaction term; for example, three atoms participate in angle interactions. The three atoms in the angle term are, therefore, each given a third of the angle energy. By averaging the energy contributions of each atom in the relevant interaction term, it will be possible to determine the total potential energy of atom *i*.

(6) $$\begin{array}{l} U^{i}=\left[\frac{1}{2}\sum_{n=1}^{N_{P}}U_{Pair}\left(r^{i}‚r^{2}\right)+\frac{1}{2}\sum_{n=1}^{N_{C}}U_{Coulomb}\left(r^{i}‚r^{2}\right)+\frac{1}{2}\sum_{n=1}^{N_{B}}U_{Bond}\left(r^{i}‚r^{2}\right)\right]\\ \;\;\;\;\;\;\;\;\;\;\;\;\;\;\;\;\;\;\;\;\;\;\;\;\;\;\;+\left[\frac{1}{3}\sum_{n=1}^{N_{A}}U_{Angle}\left(r^{i}‚r^{2}‚r^{3}\right)+\frac{1}{4}\sum_{n=1}^{N_{D}}U_{Dihedral}\left(r^{i}‚r^{2}‚r^{3}‚r^{4}\right)\right.\\ \;\;\;\;\;\;\;\;\;\;\;\;\;\;\;\;\;\;\;\;\;\;\;\;\;\;\;\left.+\frac{1}{4}\sum_{n=1}^{N_{I}}U_{Improper}\left(r^{i}‚r^{2}‚r^{3}‚r^{4}\right)\right]\;, \end{array}$$

The parameters of $N_{P}$, $r^{i}$ and $r^{2}$ indicate, respectively, the neighbors of atom *i*, the location of atom *i*, and the location of neighbor atoms in the first set of interactions, which are related to the van-der-Waals energy. The bond contribution of atom *i* with $N_{B}$ bonds relates to the third term. The same parameters, $N_{A}$, $N_{D}$ and $N_{I}$ are related to atoms that atom *i* is a part of and this is similar for other terms related to angle, dihedral, and improper interaction.

Equation (4) would be placed into Equation (3), and differentiation according to time would be performed to determine the microscopic heat flow vector ***J***.

(7) $$J\left(t\right)=\frac{1}{V}\sum_{i=1}^{N}\upsilon^{i}e^{i}+\sum_{i=1}^{N}S^{i}\cdot \upsilon^{i}\;,$$

$S^{i}$ is the stress tensor for each atom in Equation (8). The values of *x*, *y*, and *z* are assigned to the parameters *a* and *b* in order to obtain the six components of the symmetric tensor.

(8) $$\begin{array}{l} VS_{ab}^{i}=\left[\frac{1}{2}\sum_{n=1}^{N_{P}}\left(r_{a}^{i}F_{b}^{i}+r_{a}^{2}F_{b}^{2}\right)+\frac{1}{2}\sum_{n=1}^{N_{C}}\left(r_{a}^{i}F_{b}^{i}+r_{a}^{2}F_{b}^{2}\right)+\frac{1}{2}\sum_{n=1}^{N_{B}}\left(r_{a}^{i}F_{b}^{i}+r_{a}^{2}F_{b}^{2}\right)\right]\\ \;\;\;\;\;\;\;\;\;\;\;\;\;\;\;\;\;\;\;\;\;\;\;\;\;\;\;+\left[\frac{1}{3}\sum_{n=1}^{N_{A}}\left(r_{a}^{i}F_{b}^{i}+r_{a}^{2}F_{b}^{2}+r_{a}^{3}F_{b}^{3}\right)\right.\\ \;\;\;\;\;\;\;\;\;\;\;\;\;\;\;\;\;\;\;\;\;\;\;\;\;\;\;+\left.\frac{1}{4}\sum_{n=1}^{N_{D}}\left(r_{a}^{i}F_{b}^{i}+r_{a}^{2}F_{b}^{2}+r_{a}^{3}F_{b}^{3}+r_{a}^{4}F_{b}^{4}\right)\right]\\ \;\;\;\;\;\;\;\;\;\;\;\;\;\;\;\;\;\;\;\;\;\;\;\;\;\;\;+\left[\frac{1}{4}\sum_{n=1}^{N_{I}}\left(r_{a}^{i}F_{b}^{i}+r_{a}^{2}F_{b}^{2}+r_{a}^{3}F_{b}^{3}+r_{a}^{4}F_{b}^{4}\right)\right] \end{array}$$

In Equation (8), three terms make up the first bracket, where $F^{i}$ and $F^{2}$ are the forces acting on the two atoms as a result of their interaction. Furthermore, the force parameters for the angle, dihedral, and improper contacts of atoms in the following interaction sets are, likewise, the same. Furthermore, the kinetic energy is absent from the stress tensor, and the stress tensor only contains the virial terms [49].

## 3. Results and Discussion

### 3.1. Representation of Cis-1,4-Polybutadiene in MD Simulations

This work has taken into consideration a polymeric structure consisting of cis-1,4-PB crosslinked with sulfur. As was previously indicated, temperature curing and the accelerator/sulfur (A/S) ratio affect the length of the sulfur link. Sulfur combines with PB chains to create crosslinks of varying lengths, ranging from monosulfidic to polysulfidic. As a result, a particular sulfur linkage can be adjusted based on its intended use. The molecular model of each chain, including the PB and sulfur chains, was generated by Moltemplate software (version 2.20.3, Andrew Jewett, Los Angeles, CA, USA) [50].

**Figure 3:**
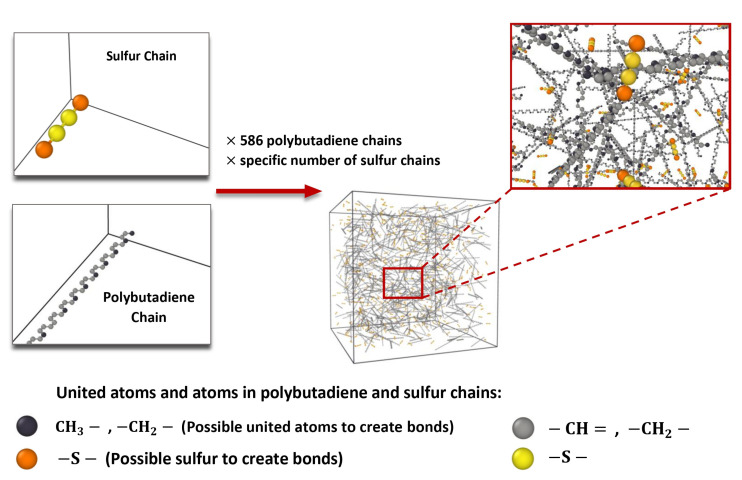
Tetra-sulfur (S4) and polybutadiene chain distribution procedure inside the simulation box.

The united-atom model is highly beneficial for researching long-chain compounds and is frequently used to represent hydrocarbons like alkanes and alkenes [51]. Some atoms in this model are merged to form a single particle, which is considered as a single unit in the simulation. Therefore, the united-atom model was used in producing the PB chain. Each of these chemical structures, including PB and sulfur, is coordinated in such a way as to prevent any overlap with one another. The cis-1,4-PB chain was generated as the molecular model of the tail group, the repeat group (body of the chain), and the head group [44]. The initial polymerization degree of each PB chain is 44 united atoms (including a carbon atom), but in the next steps this number will increase to thousands of united atoms. Subsequently, Packmol software (version 20.3.5, Leandro Martínez, Campinas, Brazil) [52] was used to randomly arrange a total of 586 PB chains and a particular number of sulfur chains (based on the related investigation’s purpose) inside a box, as displayed in Figure 3. When applying periodic conditions, it is necessary to create the related periodic cubic box large enough to prevent chain copies from interacting with each other. In Packmol, a tolerance factor of 2.0 Å was considered. In all of the following simulations, the initial simulation cell dimensions were set to a cubic box with a side length of 275 Å and a volume of 20,796.875 nm^3^.

**Figure 4:**
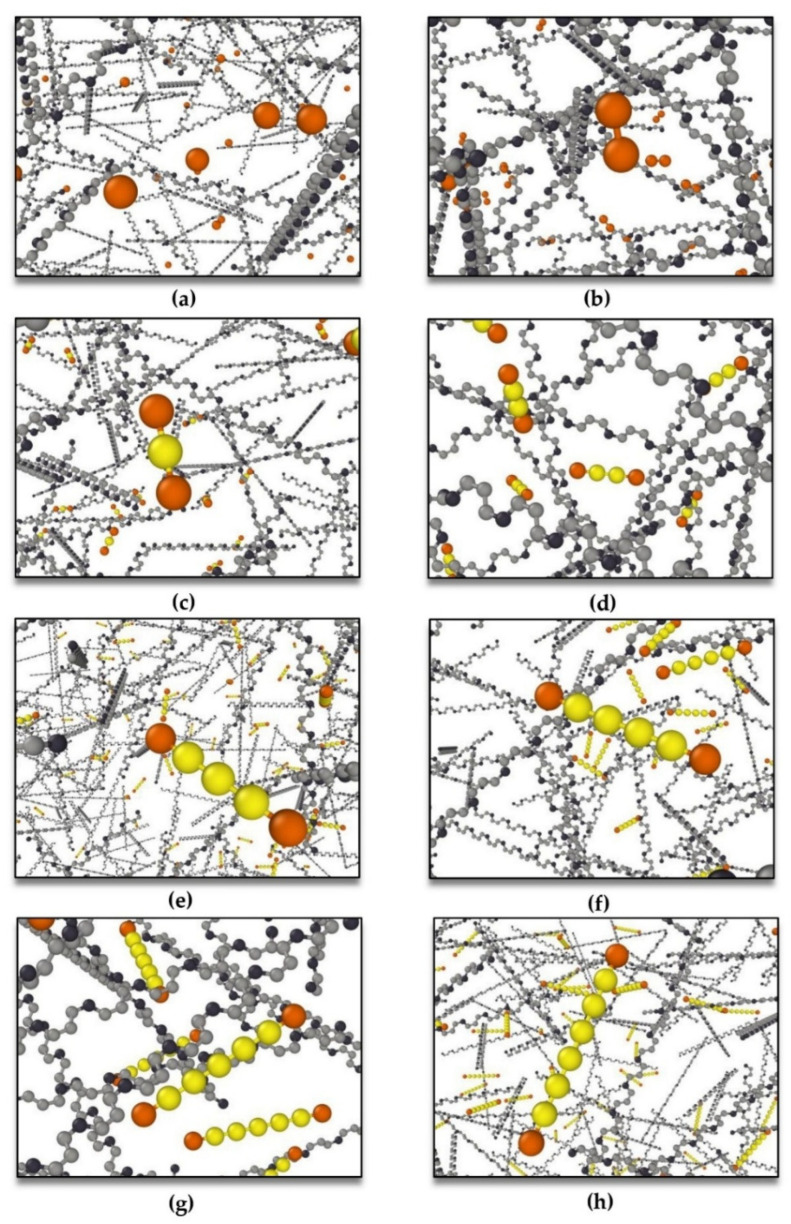
Different lengths of sulfur chains randomly distributed in the polymeric matrix: (**a**) mono-sulfur (S1), (**b**) di-sulfur (S2), (**c**) tri-sulfur (S3), (**d**) tetra-sulfur (S4), (**e**) penta-sulfur (S5), (**f**) hexa-sulfur (S6), (**g**) hepta-sulfur (S7), and (**h**) octa-sulfur (S8) were considered in eight distinct models. (Color indicators for atoms and united atoms are similar to those in Figure 3).

Different lengths of sulfur chains from mono-sulfur (S1) to octa-sulfur (S8) were taken into account in eight distinct configurations, as illustrated in Figure 4.

The LAMMPS software program (version 3 Mar 2020, Sandia National Laboratories, Albuquerque, NM, USA) [53] was utilized for all simulations. In every simulation, a united-atom force field was used. In order to improve computational efficiency and avoid explicitly representing hydrogen atoms with high-frequency movements in the structure, the –CH=, –CH_2_–, and CH_3_– groups were regarded as the “united atom”. For every simulation, the cutoff distance was established at 10 Å. Van-der-Waals interactions were used to represent non-bonded interactions using the Lennard-Jones potential. As not all “pair_styles” commands accept the “mix command” in LAMMPS, and some mix settings are not reachable for specific pair_styles, a “mixed geometric equation” was utilized to represent missing parameters for the Lennard-Jones potential in order to model all conceivable interactions [45]. One approach to validating the potential function is to compare the results of the MD simulation with experimental data. In [54], the same potential functions and parameters were utilized, and the results of the simulations were appropriately in line with the experiments, which indicates that the relevant potential functions and parameters have been considered appropriately. Table 1 provides an overview of the Lennard-Jones potential parameters as well as other force fields used in the MD simulations. Additionally, other parameters and force fields utilized in this work were presented in Table 1 of ref. [44].

The Moltemplate program was utilized to derive the parameters of the united-atom force field (OPLS-UA), which were then applied for bond stretching, van-der-Waals, dihedral, and angle interactions. In this research, a part of the dihedral and angle interactions was not taken into account because parameters of the united-atom force field do not exist for these combinations. Additionally, in every simulation, coefficients for the “special_bonds” command in LAMMPS [45] were set to zero.

**Figure 5:**
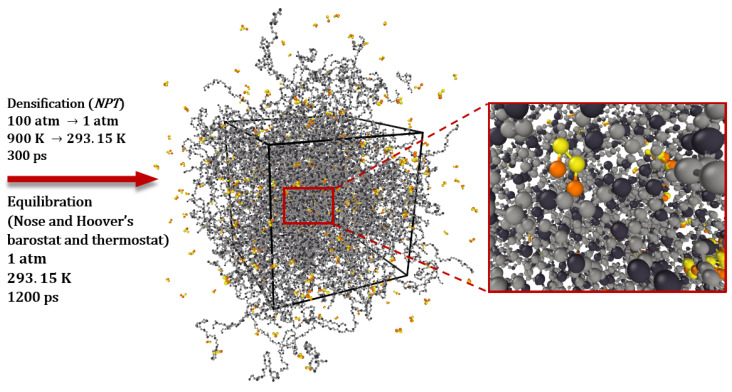
Molecular model of polybutadiene crosslinked with sulfur after densification and equilibration by Nose and Hoover’s barostat and thermostat. (Color indicators for atoms and united atoms are similar to those in Figure 3).

After the distribution of the PB and sulfur chains in the periodic supercell, as shown in Figure 3, the densification process is required. The polymeric model structure was gradually compressed using the *NPT* ensemble, as illustrated in Figure 5, from high temperatures starting at 900 K and high pressures of 100 atm to the normal temperature of 293.15 K and a normal pressure of 1 atm. This procedure was carried out once, for 300 ps, at a time step of 0.3 fs.

The next step is applying Nose and Hoover’s barostat and thermostat [55,56] to obtain a realistic density, i.e., an equilibrated density of the polymeric model structure at a particular temperature and pressure. Damping parameters of 1000 and 30 time steps for the barostat and thermostat were used, respectively. Therefore, the polymeric model system was equilibrated for 1200 ps with a time step of 0.3 fs in an *NPT* ensemble at a normal temperature of 293.15 K and a normal pressure of 1 atm.

Adopted force field parameters need to be described for the newly formed polymeric structure that is expected to be created following the crosslinking process, once the structure has stabilized. Combined atoms of –CH_2_– have the ability to form bonds with competent sulfur atoms in PB chains. Following the formation of a link, the mass of the new atoms would vary as –CH_2_– in the PB chains changed into C or –CH–. Furthermore, by joining chains together, the polymerization degree of PB chains (which currently stands at 44 united atoms) will rise to thousands of atoms. Consequently, –CH_3_ united atoms, which have located at the ends of PB chains, will convert into –CH_2_– united atoms when PB chains connect to each other. As a result, the mass of these newly created united atoms is defined for the structure. Furthermore, new bond types are also defined for these newly expected bonds. Moreover, new force fields for the crosslinked structure were introduced in Table 1.

Furthermore, a specific reaction radius (*R*_min_) of 10 Å is considered for the crosslinking setting simulations. The ratio is the other crucial quantity that has to be defined; in LAMMPS instructions, it is introduced as the “prob fraction” [45]. Since the value of the fraction parameter has been considered to be 1, all of the labeled sulfur will be able to form bonds with possible united atoms in PB chains.

**Figure 6:**
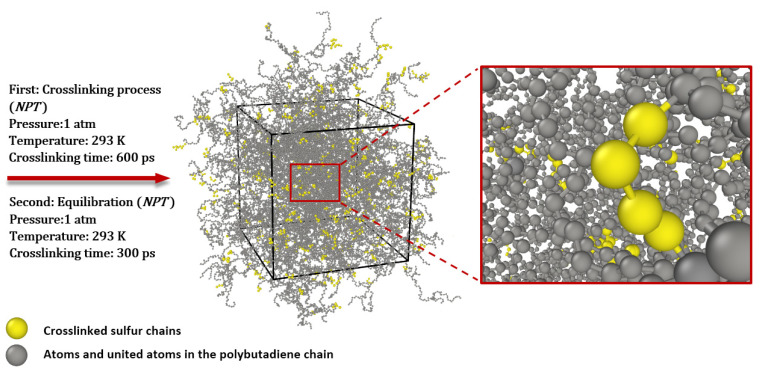
Molecular model of polybutadiene crosslinked with sulfur after crosslinking process and equilibration by Nose and Hoover’s barostat and thermostat.

The system is prepared to begin the crosslinking process with an *NPT* ensemble at a standard temperature of 293.15 K and pressure of 1 atm once these pre-crosslinking parameters are applied. After 600 ps, the structure reached saturation, and every competent sulfur atom formed bonds with other capable atoms in PB chains, as shown in Figure 6. After the crosslinking process, the system reached the potential minimization concerning temperature and density after equilibrating for 300 ps with an *NPT* ensemble at standard temperature 293.15 K and standard pressure 1 atm. At this stage, the structure is well-prepared for obtaining the glass transition temperature; therefore, by considering various temperatures in the *NPT* ensemble at a normal pressure of 1 atm, the correlation between the density of the structure and temperatures is obtained.

Finally, the normalized heat flux autocorrelation function must be computed in order to derive the heat conductivity. This can be carried out in the MD simulations by applying sequential ensembles, such as *NVT* and *NVE*, to the periodic box that holds the crosslinked polymeric framework.

After the crosslinking process and equilibration of the density, the energy of the polymeric model structure will be equilibrated. Consequently, the system is simulated in an *NVT* ensemble with a time step of 0.3 fs at a specific temperature and a simulation time of 1200 ps. The *NVT* ensemble is applied once or twice to crosslinked polymeric frameworks. Therefore, a realistic molecular model structure of crosslinked PB is produced using the above-mentioned approach.

Using the Langevin thermostat [57], an *NVE* ensemble was conducted to derive the heat flux autocorrelation function. The heat flux in each direction was then estimated as three components at each time step. For every correlation interval, the thermal conductivity was obtained by using the Green–Kubo equation [46]. The *NVE* ensemble was applied with a simulation time of around 1900 ps and a correlation length of 21 ps.

The current study investigated the effects of various sulfur chain lengths, the quantity of sulfur in the polymeric structure, and the degree of crosslinking of sulfur on glass transition temperature and thermal conductivities.

### 3.2. Cis-1,4-Polybutadiene Crosslinked with Randomly Distributed Sulfur Chains of Different Lengths with a Constant Degree of Crosslinking in All Models

In the first step, eight distinct crosslinked polymeric models composed of PB and different lengths of sulfur chains from mono-sulfur (S1) to octa-sulfur (S8) were generated, respectively, as demonstrated in Figure 4. The PB matrix in these eight models comprises 25,784 united atoms (65,632 atoms) with a constant molar mass of 349,858.41 g/mol. The number of sulfur chains is 396 in all of the following models; consequently, the degree of crosslinking is also constant for all of these eight models; however, the molar mass of sulfur varies in different structures, as summarized in Table 2.

#### 3.2.1. Determination of Thermal Conductivity with Constant Crosslinking Degree of Sulfur Bridges

**Figure 7:**
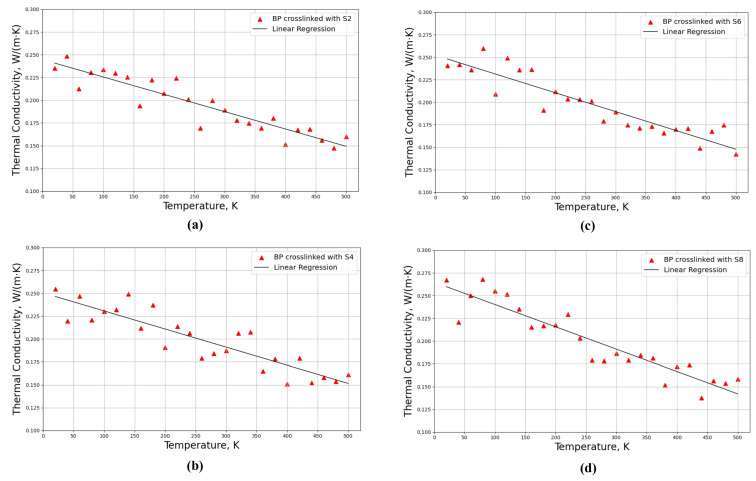
Thermal conductivities for four crosslinked polymeric models composed of randomly distributed different lengths of sulfur chains in the PB matrix when the degree of crosslinking is constant for all models for different temperatures from 20 K to 500 K; PB crosslinked with (**a**) di-sulfur (S2), (**b**) tetra-sulfur (S4), (**c**) hexa-sulfur (S6), and (**d**) octa-sulfur (S8).

Thermal conductivities for four distinct crosslinked polymeric structures of these models, including PB crosslinked with di-sulfur (S2), tetra-sulfur (S4), hexa-sulfur (S6), and octa-sulfur (S8), were investigated based on the models shown in Figure 4b,d,f,h, respectively. Thermal conductivities were investigated for different temperatures from 20 K to 500 K with a step of 20 K and a constant pressure of 1 atm, as can be observed in Figure 7.

As can be observed in Figure 7, the thermal conductivities of a crosslinked structure decrease with increasing temperature for all models. The phonon contribution, which is influenced by intermolecular interactions, chain flexibility, and molecular structure, is primarily responsible for the heat conductivity of polymers. Sulfur increases the stiffness and mobility of the polymer chains in polybutadiene, enhancing phonon transmission and thermal conductivity [44]. However, when the temperature increases, the thermal vibrations of the atoms and molecules become more intense, resulting in more scattering and attenuation of the phonons, which consequently decreases the thermal conductivity. Additionally, the thermal expansion of the polymer caused by the increased temperature also decreases the density and enhances the free volume, which further reduces the thermal conductivity [58].

The linear regression line was used to estimate the approximate thermal conductivity at any given temperature within the range of the data. Moreover, by considering the regression line, the thermal conductivity increases by increasing the length of the sulfur bridges from S2 to S8, which can be obtained through the intercept value of the regression line. For the crosslinked model composed of different lengths of sulfur, including S2, S4, S6, and S8, the intercepts of the regression lines are 0.244, 0.250, 0.253, and 0.265 W/(m·K), respectively. Therefore, the thermal conductivity increased with increasing the length of the sulfur chains and molar weight of the crosslinker, as longer chains enhanced the heat transfer between the crosslinked segments.

In addition, the thermal conductivities of these eight models with different lengths of sulfur atoms from S1 to S8 in normal conditions at a temperature of 293.15 K and a pressure of 1 atm were investigated when the degree of crosslinking was considered constant. As expected, the thermal conductivities tend to increase as the length of sulfur chains increases, as summarized in Table 3.

#### 3.2.2. Determination of Glass Transition Temperature with Constant Crosslinking Degree of Sulfur Bridges

**Figure 8:**
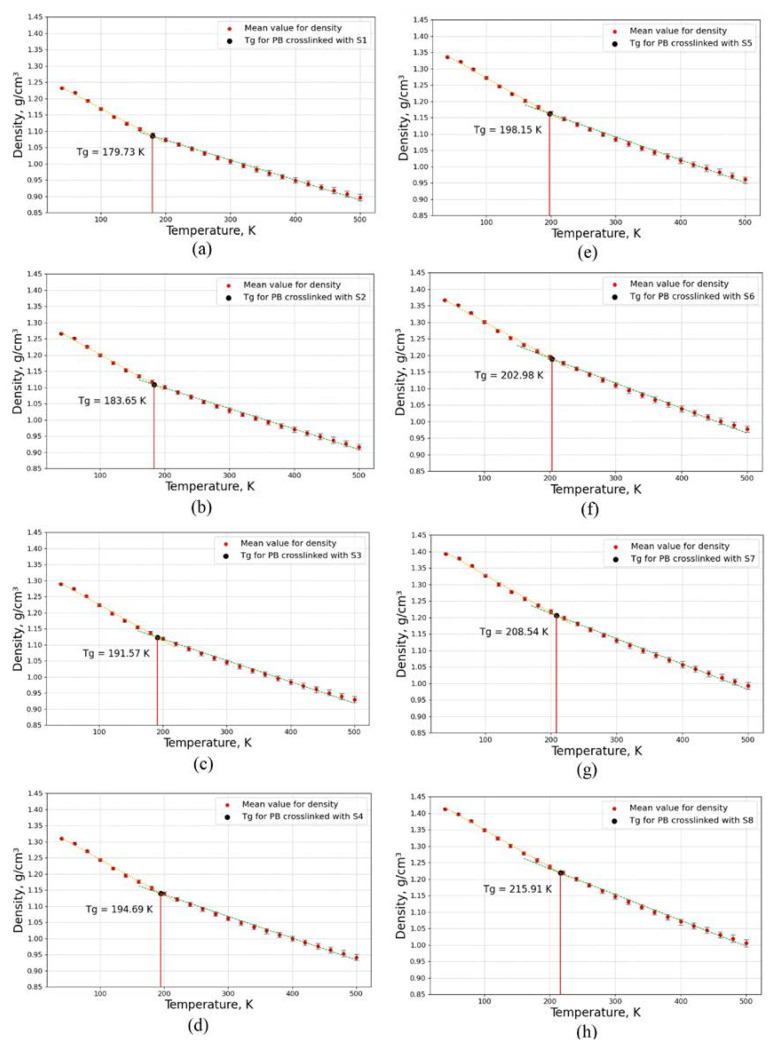
Glass transition temperature for crosslinked PB models composed of randomly distributed different lengths of sulfur chains when the degree of crosslinking is constant for all models: PB crosslinked with (**a**) mono-sulfur (S1), (**b**) di-sulfur (S2), (**c**) tri-sulfur (S3), (**d**) tetra-sulfur (S4), (**e**) penta-sulfur (S5), (**f**) hexa-sulfur (S6), (**g**) hepta-sulfur (S7), and (**h**) octa-sulfur (S8).

In this step, the glass transition temperatures (*T*_g_) of eight models, according to Figure 4, were studied by MD simulation when the degree of crosslinking was considered constant for all of these models. Glass transition temperatures were obtained by evaluating the density for different temperatures from 40 K to 500 K with a step of 20 K and a constant pressure of 1 atm, as can be observed in Figure 8.

**Figure 9:**
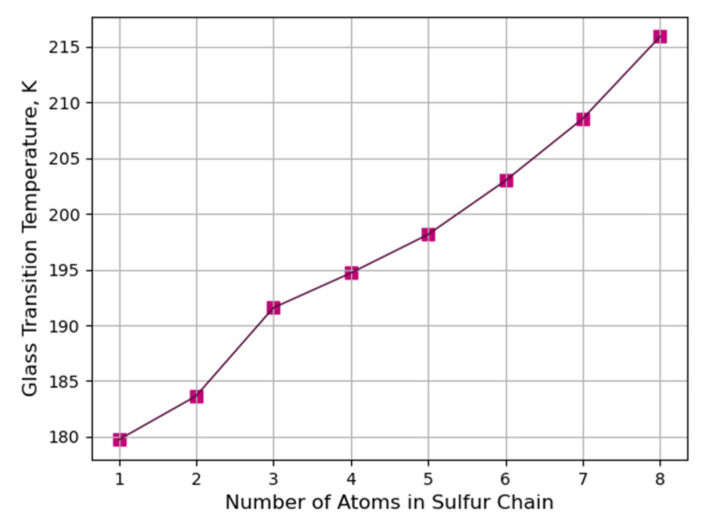
Glass transition temperature as a function of sulfur atoms in the crosslinked structure when the degree of crosslinking is constant for eight models.

According to Figure 8a–h, the glass transition temperature of the crosslinked PB increases as the length of the sulfur chains and molar mass of the sulfur increase. The glass transition temperature is 179.73 K when the crosslinked structure is composed of mono-sulfur (S1); however, the glass transition temperature will be increased to 215.91 K when the crosslinked structure is composed of octa-sulfur (S8), as displayed in Figure 9.

As the plots in Figure 8 indicate, the density of the crosslinked PB increases as the length of the sulfur chain increases. This is because longer sulfur chains have more mass and packing efficiency than shorter ones, which increase the molar weight and compactness of the network.

Moreover, the slope of the density–temperature curves changes at *T*_g_, indicating a change in the thermal expansion coefficient in Figure 8. The slope is steeper below the *T*_g_, meaning that the crosslinked PB contracts more when cooled in the glassy state. The slope is flatter above the *T*_g_, meaning that the crosslinked PB expands less when heated in the rubbery state. This is because the segmental mobility and free volume of the crosslinked PB increase above *T*_g_, which reduces the thermal contraction and expansion.

Figure 9 demonstrates the glass transition temperature as a function of sulfur atoms in the crosslinked PB when the degree of crosslinking is constant for all of these models; the molar weight of sulfur thus increases by increasing the lengths of the sulfur chains.

As can be observed in Figure 9, the glass transition temperature increases with the increasing molar mass of sulfur and length of sulfur chains, due to the free volume fraction and the molecular chain motion of the material. As the molar weight of sulfur increases, the free volume fraction decreases, resulting in a more rigid material with less mobility and flexibility. Consequently, the material requires more thermal energy to overcome the intermolecular forces and undergo the glass transition to transfer from the rubbery state to the glassy state, and vice versa.

### 3.3. Polybutadiene Crosslinked with Randomly Distributed Sulfur of Different Lengths with Constant Molar Mass of Sulfur in All Models

In the second step, eight separate crosslinked polymeric models consisting of PB and various lengths of sulfur chains from mono-sulfur (S1) to octa-sulfur (S8) were produced, respectively, as shown in Figure 4. The amount of sulfur utilized during the vulcanization process should be optimized to achieve the desired balance of mechanical properties, thermal stability, and aging resistance for a particular application. Increasing the amount of sulfur used in the vulcanization process can lead to an increase in crosslink density, which can result in improved mechanical properties such as tensile strength and hardness. Nevertheless, excessive amounts of sulfur can result in over-crosslinking, which may cause the material to become brittle and lose its elasticity [59]. For producing PB crosslinked with sulfur in [54], the amount of sulfur has been considered to be 2.8 phr (parts per hundred of rubber), according to the desired purpose.

In the current study, the amount of sulfur and PB were considered to be 3.5 phr and 96.5 phr, respectively, based on the desired mechanical purpose for these eight different crosslinked structures, as summarized in Table 4. Similar to Section 3.2, the PB matrix in all of these eight models has a constant molar mass of 349,858.41 g/mol and is made up of 586 chains and 25,784 united atoms (65,632 atoms). The degree of crosslinking varies according to the number of sulfur chains in each model, while the molar mass of sulfur stays almost constant for all configurations.

#### 3.3.1. Determination of Thermal Conductivities with Constant Molar Mass of Sulfur

**Figure 10:**
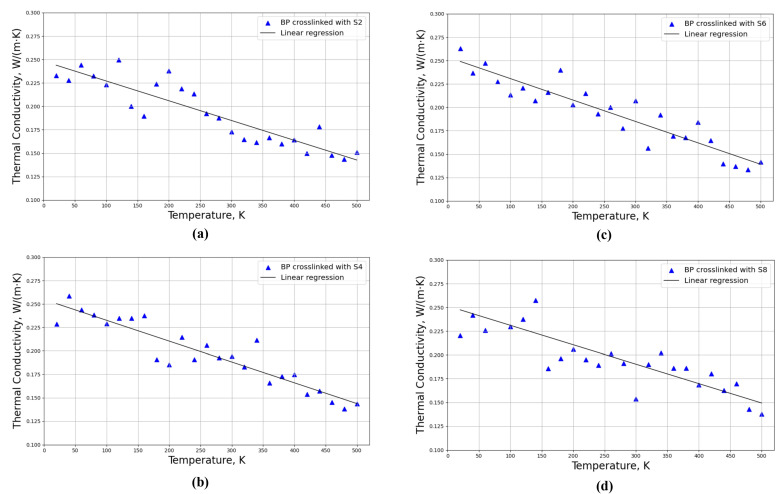
Thermal conductivities for four crosslinked polymeric models with the same molar mass of sulfur for different temperatures from 20 K to 500 K; PB crosslinked with (**a**) di-sulfur (S2), (**b**) tetra-sulfur (S4), (**c**) hexa-sulfur (S6), and (**d**) octa-sulfur (S8).

Thermal conductivities for four distinct crosslinked polymeric structures, including PB crosslinked with a constant molar mass of sulfur, including di-sulfur (S2), tetra-sulfur (S4), hexa-sulfur (S6), and octa-sulfur (S8), were investigated based on the models shown in Figure 4b,d,f,h, respectively. Thermal conductivities were investigated for different temperatures from 20 K to 500 K with a step of 20 K and a constant pressure of 1 atm, as can be observed in Figure 10.

As can be observed from Figure 10, the thermal conductivity of all models decreases with increasing temperature, similar to Figure 7. The increase in temperature induces more disorder and randomness in the polymeric structure, which also increases phonon scattering and reduces thermal conductivity. This effect may be more significant in the randomly crosslinked model, where the crosslinking distribution is more heterogeneous and the chain conformation is more complex. For instance, there are a few points with a high deviation from the regression line; as can be observed in Figure 10a, the thermal conductivity at *T* = 140, 160, and 300 K have a high deviation from the regression line. At these points, the equilibration failed for the thermal conductivity along all three axes. One possible reason for the anisotropy of thermal conductivity in the crosslinked polybutadiene structure is that the orientation of the sulfur chains affects the phonon transport in different directions. Phonons are the main carriers of heat in polymers, and their mean free path and scattering rate depend on the molecular structure and alignment. If the sulfur chains are aligned more parallel to the *x* and *y* axes, then the phonon transport along those directions will be more efficient and the thermal conductivity will be higher. However, if the sulfur chains are more perpendicular to the *z* axis, then the phonon transport along that direction will be more hindered and the thermal conductivity will be lower. Moreover, the simulation results are in line with the experimental studies in [54] for PB rubber volcanized by 2.8 phr of sulfur which has been studied in the temperature range between 273.15 K and 333.15 K; however, in the current work, as PB is crosslinked with 3.5 phr sulfur content and there are some variations in the lengths of sulfur for each structure, some differences in thermal conductivity are obvious.

For the crosslinked model composed of different lengths of sulfur, including S2, S4, S6, and S8, the intercepts of the regression lines are 0.248, 0.254, 0.254, and 0.252 W/(m·K), respectively, which shows that the trend in changes in thermal conductivities is almost the same for different crosslinked models composed of different lengths of sulfur. The degree of crosslinking and length of sulfur chains are two factors that influence thermal conductivity by influencing the molecular structure.

In addition, based on Table 5, the thermal conductivities of these eight models with different lengths of sulfur chains from S1 to S8 in normal conditions at a temperature of 293.15 K and a pressure of 1 atm are investigated when the molar mass of sulfur is constant for all eight models and the degree of crosslinking is reduced by increasing the length of the sulfur bridges. As expected, according to the results in Figure 10, the thermal conductivities are almost constant.

#### 3.3.2. Determination of Glass Transition Temperature with a Constant Molar Mass of Sulfur

**Figure 11:**
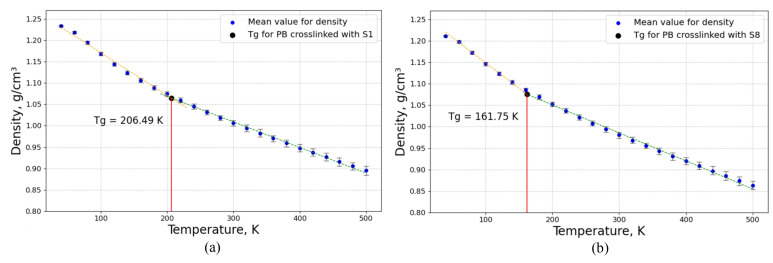
Glass transition temperature for crosslinked PB models composed of randomly distributed different lengths of sulfur chains of (**a**) mono-sulfur (S1) and (**b**) octa-sulfur (S8) when the molar mass of sulfur is constant but the degree of crosslinking changes. (The glass transition temperature figure for all of these eight models is in the Supplementary Materials).

In this step, the glass transition temperatures (*T*_g_) of eight models, according to Figure 4, were studied using MD simulation when the molar mass of sulfur is constant and the length of sulfur bridges changes for all of these models. The glass transition temperature was obtained by evaluating density at different temperatures, from 40 K to 500 K, with a step of 20 K and a constant pressure of 1 atm. Figure 11 displays the glass transition temperature for crosslinked PB models composed of randomly distributed sulfur chains of mono-sulfur (S1) and octa-sulfur (S8).

**Figure 12:**
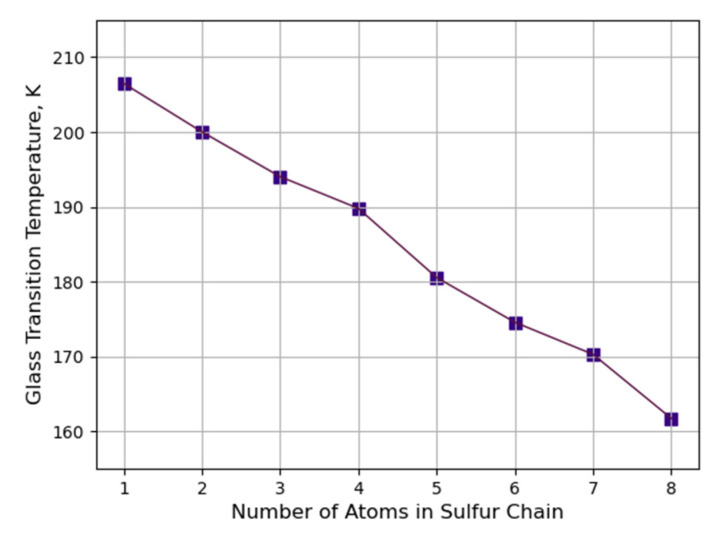
Glass transition temperature as a function of sulfur atoms in the crosslinked PB for eight models when the molar mass of sulfur is constant and the degree of crosslinking is decreasing.

According to Figure 11, the glass transition temperature of the crosslinked PB decreases with increasing the length of the sulfur chains and decreasing the degree of crosslinking. The reason that the glass transition temperature decreases with the decreasing degree of crosslinking is due to the free volume fraction and the molecular chain motion of the material. The degree of crosslinking is the primary factor that influences the free volume fraction of the material in both the rubbery and glassy states. As the number of crosslinks increases, the free volume fraction decreases, resulting in a more rigid and stable material. Consequently, the material requires more thermal energy to overcome the intermolecular forces and transition from the rubbery state to the glassy state, or vice versa. In addition, the lower the crosslinking degree, the higher the self-diffusion coefficient of the material, indicating that the molecular chain has more mobility and flexibility. Thus, the crosslinked material requires less energy to overcome the intermolecular forces and undergo the glass transition [60,61]. Consequently, the glass transition temperature decreases with the decrease in the crosslinking degree, as shown in Figure 12.

The glass transition temperature as a function of sulfur atoms in the crosslinked PB is displayed in Figure 12, when the molar mass of the sulfur is constant for all eight models and the degree of crosslinking is decreasing while the lengths of the sulfur chains are increasing. The glass transition temperature (*T*_g_) values for different PB samples with varying configuration isomeric compositions were studied by Makhiyanov et al. [62], in which study the range of glass transition temperature is comparatively in line with our research.

## 4. Conclusions

The influence of the length of the sulfur chains, the molar weight of the crosslinker, and the degree of crosslinking on the glass transition temperature and the final values of thermal conductivities were studied for a wide range of temperatures. In the first step, the degree of crosslinking is considered constant for the eight simulation models composed of mono-sulfur (S1) to octa-sulfur (S8), while the molar mass of the sulfur is increasing. The thermal conductivities of the crosslinked structure decrease with an increase in temperature for each model, due to more scattering and attenuation of the phonons, along with enhancement of the free volume. Moreover, by increasing the lengths of the sulfur chains and the molar weight of the crosslinker, thermal conductivity increases at a constant temperature. The glass transition temperature and density of the crosslinked structure are enhanced by increasing the molar mass and length of the sulfur chains, owing to decreasing the free volume fraction and the mobility of the molecular chains. Consequently, the material requires more thermal energy to overcome the intermolecular forces and undergo the glass transition. In the second step, the molar weight of sulfur is considered constant for eight models; therefore, the degree of crosslinking decreases with the increase in the lengths of the sulfur chains. The thermal conductivities of the crosslinked structure decrease with the increase in the temperature for each model due to the intensive vibration of atoms and molecules, which in turn results in more phonon scattering. Moreover, by increasing the lengths of sulfur chains and thus decreasing the degree of crosslinking, the trend in changes in thermal conductivities is almost the same for all of these models, so thermal conductivity is almost constant for a specific temperature. In addition, the glass transition temperature and density of the crosslinked structure decrease by decreasing the degree of crosslinking, due to the free volume fraction and the molecular chain motion of the material. By increasing the degree of crosslinking, the free volume fraction decreases, resulting in a more rigid and stable material. Consequently, the material requires more thermal energy to overcome the intermolecular forces. In addition, by decreasing the crosslinking degree, the flexibility of the chains increases, due to the increasing self-diffusion coefficient. Thus, the crosslinked material requires less energy to undergo the glass transition.

## Figures and Tables

**Table 1:** Force field parameters used in MD simulations. The usual factor of ½ in stretching and bending interactions is included in $k_{a}$ and $k_{b}$.

Force Field Parameters for Polybutadiene Crosslinked with Sulfur		
$U_{LJ}=4\epsilon \left[{\left(\frac{\sigma }{r}\right)}^{12}-{\left(\frac{\sigma }{r}\right)}^{6}\right]$	$\epsilon \;\left[\frac{kcal}{mol}\right]$	$\sigma \left[Å\right]$
$\left({CH}_{3}-\right)‚(-S-)$	0.209	3.723
$\left({-CH}_{2}-\right)‚(-S-)$	0.172	3.723
$\left(-CH=\right),(-S-)$	0.170	3.673
$\left(-CH<\right),(-S-)$	0.128	3.525
$\left(-S-\right),(-S-S-)$	0.250	3.550
$\left(-S-\right),(-S-)$	0.250	3.550
$U_{bond}=k_{b}{\left(r-r_{0}\right)}^{2}$	$k_{b}\left[\frac{kcal}{mol\;{Å}^{2}}\right]$	$r_{0}\left[Å\right]$
${CH}_{3}-CH=$	317.0	1.500
$-{CH}_{2}-C\left(>C<\right)$	260.0	1.526
$=CH-C\left(>C<\right)$	317.0	1.500
$-S-C\left(>C<\right)$	222.0	1.810
$U_{angle}=k_{a}{\left(\theta -\theta_{0}\right)}^{2}$	$k_{a}\left[\frac{kcal}{mol\;{rad}^{2}}\right]$	$\theta_{0}\left[degrees\right]$
${CH}_{3}-CH=CH-$	70.0	118.0
$-S-S-C\left(>C<\right)$	68.0	103.7
$C\left(>C<\right)-S-C\left(>C<\right)$	62.0	98.9
$C\left(>C<\right)-S-CH<$	62.0	98.9
$-{CH}_{2}-C\left(>C<\right)-S-$	---	---
$=CH-C\left(>C<\right)-S-$	---	---
$=CH-C\left(>C<\right)-{CH}_{2}-$	---	---
$=CH-CH\left(-{CH}_{2}\right)-S-$	---	---

**Table 2:** Eight different crosslinked structures with different type of sulfur chains and similar degree of crosslinking.

Type of Sulfur	Number of Sulfur Chains	Number of Sulfur Atoms in the Structure	PB Content (phr)	Sulfur Content (phr)	Molar Mass of Sulfur (g/mol)
Mono-sulfur (S1)	396	396	96.5	3.5	12,697
Di-sulfur (S2)	396	792	93.0	7.0	25,395
Tri-sulfur (S3)	396	1188	89.5	10.5	38,093
Tetra-sulfur (S4)	396	1584	86.0	14.0	50,790
Penta-sulfur (S5)	396	1980	82.5	17.5	63,488
Hexa-sulfur (S6)	396	2376	79.0	21.0	76,186
Hepta-sulfur (S7)	396	2772	75.5	24.5	88,884
Octa-sulfur (S8)	396	3168	72.0	28.0	101,581

**Table 3:** Thermal conductivities of crosslinked PB with different lengths of sulfur chains from S1 to S8 in normal conditions at a temperature of 293.15 K and a pressure of 1 atm when the degree of crosslinking is constant.

Number of Sulfur Atoms	S1	S2	S3	S4	S5	S6	S7	S8
Thermal Conductivity (W/m·K)	0.176	0.189	0.185	0.189	0.195	0.201	0.204	0.213

**Table 4:** Eight different crosslinked structures with a constant amount of 3.5 phr sulfur content and 96.5 phr PB content.

Type of Sulfur	Number of Sulfur Chains	Number of Sulfur Atoms in the Structure	Molar Mass of Sulfur (g/mol)
Mono-sulfur (S1)	396	396	12,697
Di-sulfur (S2)	198	396	12,697
Tri-sulfur (S3)	132	396	12,697
Tetra-sulfur (S4)	99	396	12,697
Penta-sulfur (S5)	79	395	12,665
Hexa-sulfur (S6)	66	396	12,697
Hepta-sulfur (S7)	57	399	12,793
Octa-sulfur (S8)	50	400	12,826

**Table 5:** Thermal conductivities of crosslinked PB with different lengths of sulfur atoms from S1 to S8 in normal conditions at a temperature of 293.15 K and a pressure of 1 atm, when the molar mass of sulfur is constant.

Number of Sulfur Atoms	S1	S2	S3	S4	S5	S6	S7	S8
Thermal Conductivity (W/m·K)	0.186	0.192	0.191	0.189	0.187	0.189	0.194	0.190

## Data Availability

Data are contained within the article and Supplementary Materials.

## Supplementary Materials

The following supporting information can be downloaded at: https://www.mdpi.com/article/10.3390/polym16030384/s1, Figure S1: Glass transition temperature for crosslinked PB models composed of randomly distributed different lengths of sulfur chains when the molar mass of sulfur is constant but the degree of crosslinking changes for all models: PB crosslinked with (a) mono-sulfur (S1), (b) di-sulfur (S2), (c) tri-sulfur (S3), (d) tetra-sulfur (S4), (e) penta-sulfur (S5), (f) hexa-sulfur (S6), (g) hepta-sulfur (S7), and (h) octa-sulfur (S8).
