# Supplementary material for: Glass Transition Temperatures and Thermal Conductivities of Polybutadiene Crosslinked with Randomly Distributed Sulfur Chains Using Molecular Dynamic Simulation
Source: Polymers (Basel). 2024 Jan 30;16(3):384. doi: 10.3390/polym16030384 (PMC10857057; doi:10.3390/polym16030384)
Supplement: Supplementary file 1 [file polymers-16-00384-s001.zip › polymers-2816189-supplementary.pdf]

Supplementary

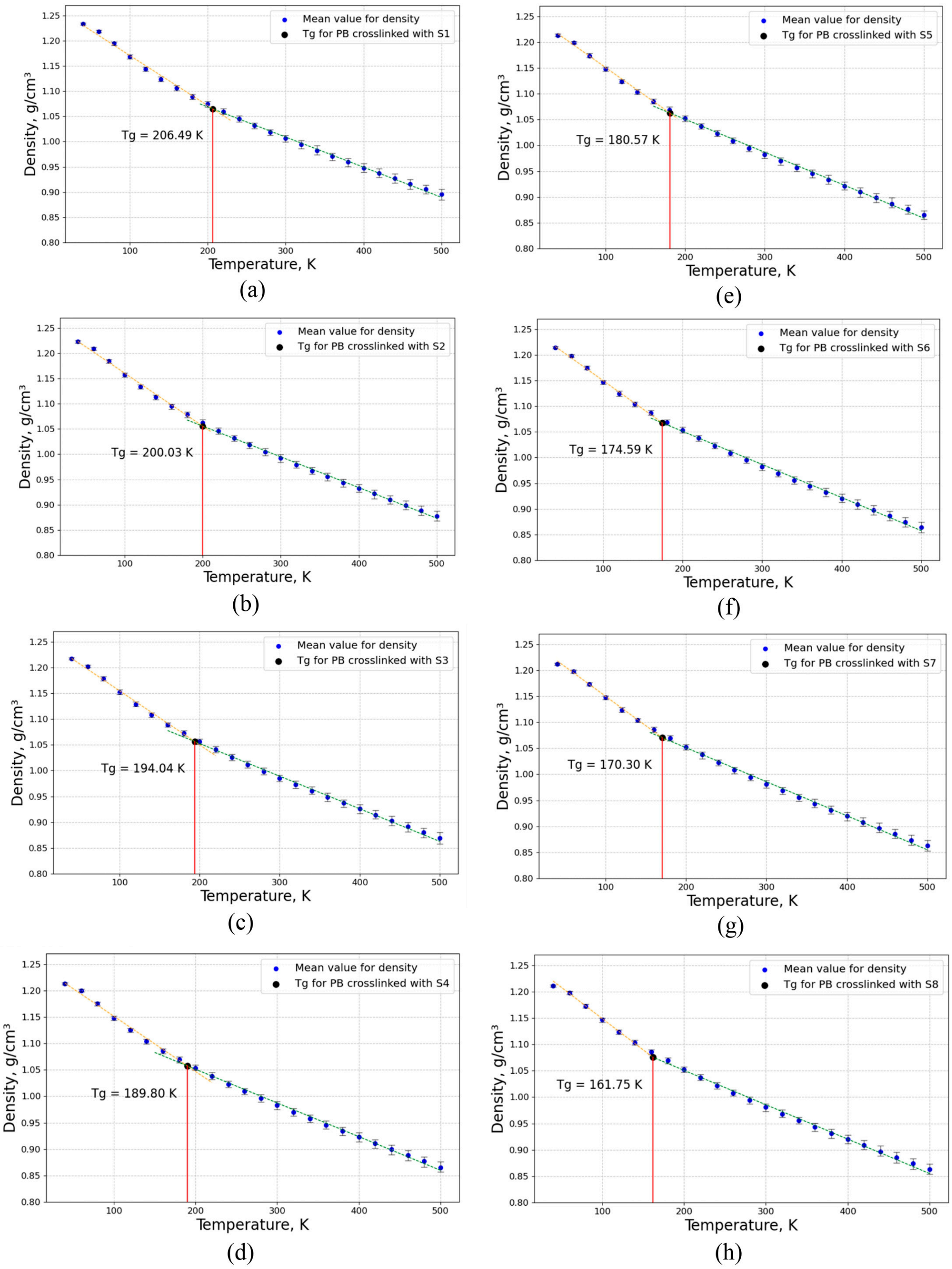

Figure S1. Glass transition temperature for crosslinked PB models composed of randomly distributed different lengths of sulfur chains when the molar mass of sulfur is constant but the degree of crosslinking changes for all models: PB crosslinked with (**a**) mono-sulfur (S1), (**b**) di-sulfur (S2), (**c**) tri-sulfur (S3), (**d**) tetra-sulfur (S4), (**e**) penta-sulfur (S5), (**f**) hexa-sulfur (S6), (**g**) hepta-sulfur (S7), and (**h**) octa-sulfur (S8).
